# Complete Genome Sequence of a Genotype G23P[37] Pheasant Rotavirus Strain Identified in Hungary

**DOI:** 10.1128/genomeA.00119-16

**Published:** 2016-03-31

**Authors:** János Gál, Szilvia Marton, Katalin Ihász, Hajnalka Papp, Ferenc Jakab, Yashpal S. Malik, Krisztián Bányai, Szilvia L. Farkas

**Affiliations:** a Department of Exotic Animal and Wildlife Medicine, Faculty of Veterinary Science, Szent István University, Budapest, Hungary; b Momentum Pathogen Discovery Group, Institute for Veterinary Medical Research, Centre of Agricultural Research, Hungarian Academy of Sciences, Budapest, Hungary; c Momentum Cancer Biomarker Research Group, Research Centre for Natural Sciences, Hungarian Academy of Sciences, Magyar tudósok körútja, Budapest, Hungary; d Virological Research Group, Szentágothai Research Centre, University of Pécs, Pécs, Hungary; e Institute of Biology, Faculty of Sciences, University of Pécs, Pécs, Hungary; f Division of Biological Standardization, Indian Veterinary Research Institute, Izatnagar, Bareilly, Uttar Pradesh, India

## Abstract

We investigated the genomic properties of a rotavirus A strain isolated from diarrheic pheasant poults in Hungary in 2015. Sequence analyses revealed a shared genomic constellation (G23-P[37]-I4-R4-C4-M4-A16-N10-T4-E4-H4) and close relationship (range of nucleotide sequence similarity: VP2, 88%; VP1 and NSP4, 98%) with another pheasant rotavirus strain isolated previously in Germany.

## GENOME ANNOUNCEMENT

Rotaviruses (RVs) are members of the family *Reoviridae*, are widely distributed, and may cause severe diarrhea in infants and young animals belonging to different mammalian and avian species (1). As of now, based on sequence and antigenic properties of the inner capsid VP6 protein, RVs have been classified into nine groups or species (designated RVA to RVI) (2, 3). RV strains possess a double-stranded RNA genome consisting of 11 segments (4). With few exceptions, all genomic segments encode a single protein, enabling the virus to express six structural and five or six nonstructural viral proteins.

RVs have been detected in many avian species, such as ducks, pheasants, chickens, turkeys, pigeons, and wild birds, in several different countries and are known to be associated with diarrhea, as well as growth retardation and runting-stunting syndrome (5, 6). Most detected avian RVs belong to group or species RVA, but RVD strains have also been seen frequently, followed by occasional infection with RVF or RVG strains (7).

In this study, we investigated the genomic properties of a pheasant RVA strain detected in Hungary in 2015. The strain, RVA/pheasant-wt/HUN/216/2015/G23P[37] (here called 216/2015), was identified in pooled stool samples from young, 7-week-old pheasant poults (*Phasianus colchicus*) with ruffled feathers, poor appetite, increased water consumption, diarrhea, and slightly increased mortality in the flock. The genome sequence of strain 216/2015 was determined applying a random primer amplification method and semiconductor sequencing (8). The complete genome sequence was assembled using the software CLC Genomics Workbench (CLC bio). Phylogenetic analysis was performed using the MEGA6 package (9). Genotyping was performed using the online tool RotaC version 2.0 (10).

The complete genome of strain 216/2015 was 18,947 bp long. The RVA genes encoding the structural proteins VP1 to VP4, VP6 and VP7, and nonstructural proteins NSP1 to NSP6 were identified (VP1, 1,089 amino acid [aa] in length; VP2, 897 aa; VP3, 829 aa; VP4, 763 aa; VP6, 397 aa; VP7, 330 aa; NSP1, 577 aa; NSP2, 315 aa; NSP3, 306 aa; NSP4, 169 aa; NSP5, 218 aa; and NSP6, 96 aa). The 5′ [5′-GGC (U/A) (U/A) (U/A) AA (A/U)-3′] and 3′ terminus sequences [5′-(A/U) U (G/A) UGACC-3′] were conserved in all genomic segments. The genotype constellation of strain 216/2015 was G23-P[37]-I4-R4-C4-M4-A16-N10-T4-E4-H4. This constellation shared several features with other pheasant RVA strains detected in the past in Germany (RVA/pheasant-tc/GER/10V0112H5/2010/G23P[37]) and Hungary (11, 12).

With the exception of the VP4 gene, the genome sequence of strain 216/2015 was similar to that of other avian RVAs and was more closely related to strain RVA/pheasant-tc/GER/10V0112H5/2010/G23P[37] (sequence similarity ranges, 88 to 98% at the nucleotide level and 91 to 99% at the amino acid level). In all gene phylogenies, strain 216/2015 clustered together with other avian RVAs; however, in the VP4 gene calculations, the Hungarian and German pheasant strains formed a monophyletic branch and appeared to be more closely related to mammalian RVAs than to avian RVAs, suggesting a distinct evolutionary history of RVs in this avian host (11).

### Nucleotide sequence accession numbers.

The genome sequence of the pheasant RVA strain 216/2015 (RVA/pheasant-wt/HUN/216/2015/G23P[37]) has been deposited to GenBank under accession numbers KU587853 to KU587863.
